# Current Tobacco Smoking, Quit Attempts, and Knowledge About Smoking Risks Among Persons Aged ≥15 Years — Global Adult Tobacco Survey, 28 Countries, 2008–2016

**DOI:** 10.15585/mmwr.mm6738a7

**Published:** 2018-09-28

**Authors:** Indu B. Ahluwalia, Tenecia Smith, René A. Arrazola, Krishna M. Palipudi, Isabel Garcia de Quevedo, Vinayak M. Prasad, Alison Commar, Kerstin Schotte, Paul David Garwood, Brian S. Armour

**Affiliations:** ^1^Office on Smoking and Health, National Center for Chronic Disease Prevention and Health Promotion, CDC; ^2^CDC Foundation, Atlanta, Georgia; ^3^Prevention of Noncommunicable Diseases, World Health Organization, Geneva, Switzerland.

Each year, tobacco use causes approximately 7 million deaths worldwide, including approximately 6 million among tobacco users and an estimated 890,000 among nonsmokers exposed to secondhand smoke (*1*). Tobacco use is a leading preventable cause of disease globally and has been determined to cause adverse health outcomes such as coronary heart disease, stroke, and multiple types of cancer, including lung cancer (*2*–*4*). Approximately 80% of the world’s 1.1 billion tobacco smokers reside in low- and middle-income countries (*4*). Some persons do not fully understand the health risks associated with tobacco smoking (*5*–*9*), and studies have indicated that increasing knowledge about the adverse health effects of smoking can contribute to decreases in smoking, increases in cessation attempts, and increases in successful cessation (*3*,*7*,*10*). CDC analyzed 2008–2016 Global Adult Tobacco Survey (GATS) data from 28 countries to assess tobacco smoking prevalence, quit attempts, and knowledge about tobacco smoking risks among persons aged ≥15 years. Across countries, the median prevalence of tobacco smoking was 22.5%, and a median of 42.5% of tobacco smokers had made a quit attempt in the preceding 12 months. The median prevalences of knowing that tobacco smoking causes stroke, heart attack, and lung cancer were 73.6%, 83.6%, and 95.2%, respectively. Implementation of proven tobacco control interventions, including strategies that increase knowledge about the health risks posed by tobacco use, might help to reduce tobacco use and tobacco-related disease, including heart disease, stroke, and lung cancer (*3*–*5*).

GATS is a nationally representative household survey of noninstitutionalized persons aged ≥15 years that uses a standard core questionnaire, sample design, and data collection methods. GATS was conducted in 28 countries during 2008–2016, with sample sizes ranging from 4,250 (Malaysia) to 74,037 (India). The median response rate was 92.0% (range = 64.4% [Ukraine] to 98.5% [Qatar]). The most recent publically available data for each country were used for analysis. Data were adjusted for nonresponse and weighted to provide nationally representative estimates for persons aged ≥15 years.

Current tobacco smokers* were defined as persons who, when asked “Do you currently smoke tobacco on a daily basis, less than daily, or not at all?” responded “daily” or “less than daily.” Tobacco smokers who made a quit attempt were defined as those who answered “yes” to the question “During the past 12 months, have you tried to stop smoking?” Knowledge that tobacco smoking causes stroke, heart attack, and lung cancer was defined as an answer of “yes” to the question “Based on what you know/believe, does smoking tobacco cause the following: Stroke (blood clots in the brain that may cause paralysis)? Heart attack? Lung cancer?” These three health outcomes were selected for analysis because they were asked by all countries as part of the core GATS questionnaire. Changes in these indictors over time were examined for eight countries with two available waves of data.

Overall country-specific prevalence estimates with corresponding 95% confidence intervals were calculated for current tobacco smoking, quit attempts, and knowledge that smoking causes stroke, heart attack, and lung cancer. Chi-squared tests were used to assess significant differences (p-value <0.05) between groups and across countries with two available waves of data. All analyses were conducted using statistical software.

Across all 28 countries, the median prevalence of current tobacco smoking was 22.5%, ranging from 3.9% (95% CI = 3.3–4.5) in Nigeria to 38.2% (95% CI = 35.7–40.8) in Greece. Among current smokers, the median prevalence of a reported past-year quit attempt was 42.5%, ranging from 14.4% (95% CI = 11.9–17.2) in China to 59.6% (95% CI = 52.4–66.5) in Senegal (Supplementary Table, https://stacks.cdc.gov/view/cdc/58990). Overall median prevalence of knowledge about adverse health outcomes caused by tobacco smoking was 73.6% for stroke (range = 27.2% in China to 89.2% in Romania), 83.6% for heart attack (range = 38.7% in China to 95.5% in Turkey), and 95.2% for lung cancer (range = 73.0% in Nigeria to 98.6% Argentina). Knowledge that smoking causes stroke (Figure 1), heart attack (Figure 2), and lung cancer (Figure 3) was significantly higher among nonsmokers than among smokers in 19, 20, and 20 countries, respectively. Eight countries with data from multiple years indicated that, in general, there were significant increases in knowledge about most indicators (Table).

**FIGURE 1:**
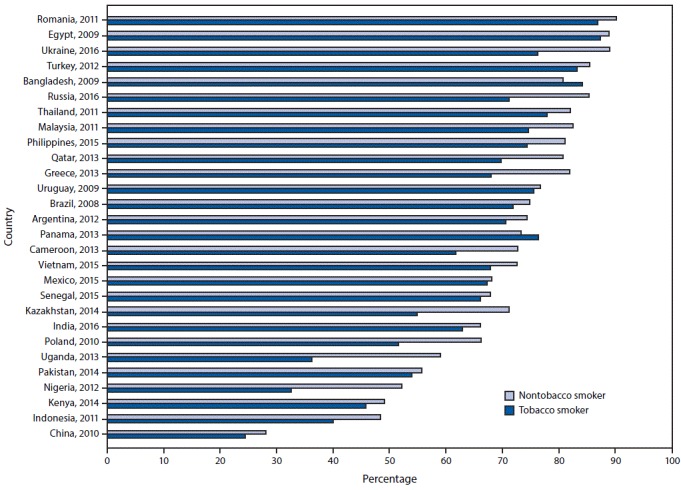
Percentage of respondents who knew that tobacco smoking causes stroke, by tobacco-smoking status and country — Global Adult Tobacco Survey, 28 countries,* 2008–2016 * Statistically significant differences between nontobacco smokers and tobacco smokers (p<0.05) occurred in Bangladesh (2009), Brazil (2008), Cameroon (2013), China (2010), Greece (2013), India (2016), Indonesia (2011), Kazakhstan (2014), Malaysia (2011), Nigeria (2012), Philippines (2015), Poland (2010), Qatar (2013), Romania (2011), Russia (2016), Thailand (2011), Uganda (2013), Ukraine (2016), and Vietnam (2015).

**FIGURE 2:**
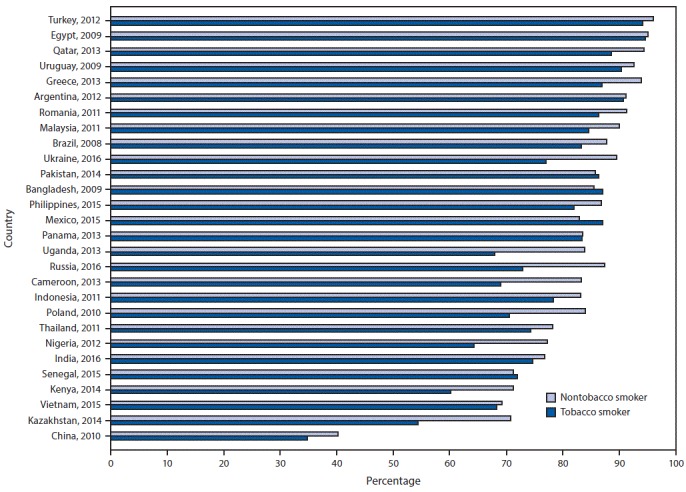
Percentage of respondents who knew that tobacco smoking causes heart attack, by tobacco-smoking status and country — Global Adult Tobacco Survey, 28 countries,* 2008–2016 * Statistically significant differences between nontobacco smokers and tobacco smokers (p<0.05) occurred in Brazil (2008), Cameroon (2013), China (2010), Greece,(2013), India (2016), Indonesia (2011), Kazakhstan (2014), Kenya (2014), Malaysia (2011), Mexico (2015), Nigeria (2012), Philippines (2015), Poland (2010), Qatar (2013), Romania (2011), Russia (2016), Thailand (2011), Turkey (2012), Uganda (2013), and Ukraine (2016).

**FIGURE 3:**
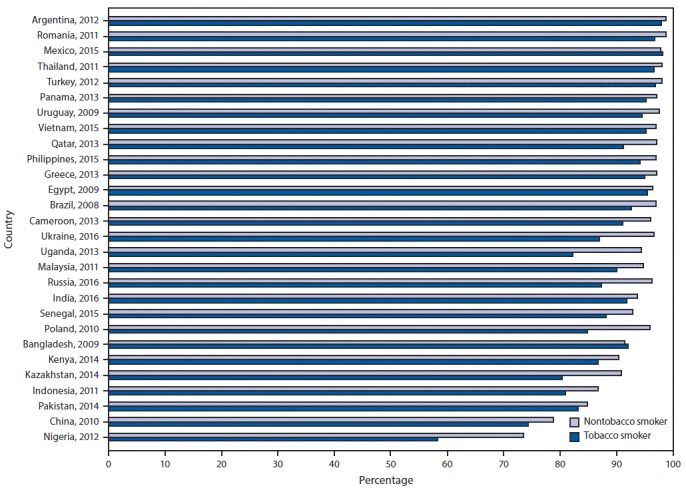
Percentage of respondents who knew that tobacco smoking causes lung cancer, by tobacco-smoking status and country — Global Adult Tobacco Survey, 28 countries,* 2008–2016 * Statistically significant differences between non-tobacco smokers and tobacco smokers (p<0.05) occurred in Brazil (2008), Cameroon (2013), China (2010), Greece (2013), India (2016), Indonesia (2011), Kazakhstan (2014), Malaysia (2011), Nigeria (2012), Philippines (2015), Poland (2010), Qatar (2013), Romania (2011), Russia (2016), Thailand (2011), Turkey (2012), Uganda (2013), Ukraine (2016), Uruguay (2009), and Vietnam (2015).

**TABLE:** Relative change* in knowledge/belief that tobacco smoking causes stroke, heart attack, and lung cancer, overall and by smoking status for countries with two waves of data — Global Adult Tobacco Survey, 2008–2017

Country, yrs of survey	Smoking status	Stroke	Heart attack	Lung cancer
India, 2009/10 and 2016/17	Overall	33.2^†^	43.2^†^	31.6^†^
	Smoker	19.9^†^	29.6^†^	18.4^†^
	Nonsmoker	10.1^†^	13.8^†^	9.4^†^
Mexico, 2009 and 2015	Overall	12.5^†^	14.9^†^	12.0^†^
	Smoker	4.8^†^	4.9^†^	4.8^†^
	Nonsmoker	1.2^†^	1.3^†^	1.1^†^
Philippines, 2009 and 2015	Overall	8.6^†^	15.0^†^	5.8^†^
	Smoker	8.6^†^	15.8^†^	5.7^†^
	Nonsmoker	3.9^†^	8.5^†^	2.0^†^
Russia, 2009 and 2016	Overall	20.6^†^	30.4^†^	13.3^†^
	Smoker	16.9^†^	22.0^†^	11.7^†^
	Nonsmoker	2.6^†^	3.0	1.0
Thailand, 2009 and 2011	Overall	1.8	−0.2	2.4^†^
	Smoker	2.0	2.2	2.0
	Nonsmoker	0.3	0.4	0.3
Turkey, 2008 and 2012	Overall	3.4^†^	1.1	4.3^†^
	Smoker	2.1^†^	0.2	2.8^†^
	Nonsmoker	1.7^†^	0.4	2.2^†^
Ukraine, 2010 and 2017	Overall	10.5^†^	10.8^†^	9.0^†^
	Smoker	9.4^†^	8.9^†^	8.3^†^
	Nonsmoker	3.6^†^	0.4	3.9^†^
Vietnam, 2010 and 2015	Overall	1.7	7.2^†^	0.1
	Smoker	10.3^†^	22.8^†^	6.9^†^
	Nonsmoker	1.1^†^	3.9^†^	0.3

* Relative change is calculated as ([percentage at second wave (t2) - percentage at first wave (t1)] / percentage at first wave [t1]) * 100.

^†^ Statistically significant, p<0.05.

## Discussion

Current tobacco smoking prevalence remains high in many of the assessed countries, and in 24 of 28 countries, fewer than half of current tobacco smokers had made a past-year quit attempt. Although knowledge about the risks posed by smoking was high across most countries, knowledge prevalence was generally lower among smokers than nonsmokers. In eight countries with two waves of available data, knowledge increased, although it varied among countries by indicator and whether or not the respondent was a smoker. Knowledge regarding the dangers of tobacco smoking is important for developing evidence-based interventions to reduce tobacco use (*2*,*7*,*9*), which is critical to reducing premature mortality from noncommunicable diseases (*4*). Opportunities exist for countries to increase tobacco cessation and prevent initiation through proven strategies that warn about the dangers of tobacco smoking and promote the benefits of quitting (*3*–*5*).

Implementation of the World Health Organization (WHO) Framework Convention of Tobacco Control (FCTC)^†^ and MPOWER^§^ might reduce tobacco use. The WHO FCTC calls for its Parties to adopt and implement measures to reduce tobacco use. MPOWER is a package of six evidence-based tobacco demand reduction policies developed by WHO to help implement the WHO FCTC at the country level. This study found that knowledge about the risks for tobacco smoking was significantly lower among smokers than among nonsmokers in the majority of countries; this lack of knowledge might contribute to fewer smokers making a quit attempt. One of the MPOWER measures includes warning about the dangers of tobacco via graphic health warning labels and mass media campaigns to increase knowledge that smoking causes chronic disease. Adopting these strategies to increase knowledge about the risks for smoking could help decrease tobacco smoking prevalence, increase cessation attempts, and increase successful cessation (*3*,*7*).

The WHO FCTC and MPOWER demand-reduction package outlines an evidence base for countries to use to respond to the tobacco epidemic by implementing specific programs and policies (*3*–*5*). As of January 2018, 181 countries and members have ratified the WHO FCTC, including all 28 countries included in this report. In 2015, the United Nations General Assembly, including countries that were signatories of WHO FCTC, adopted the 2030 Agenda for Sustainable Development, which includes multiple development goals; one of these (Goal 3) focuses specifically on improving health.^¶^ Two targets related to Goal 3 include strengthening the implementation of the WHO FCTC in all countries (Target 3.A.1) and reducing noncommunicable disease mortality by one third by 2030 (Target 3.4). Countries monitor both targets by assessing reductions in tobacco use.

The findings in this report are subject to at least four limitations. First, data were self-reported, which might result in misreporting of smoking behavior. Second, only a limited number of countries were assessed; thus, the findings in this report might not be generalizable to all countries. Third, the data were collected in different years, which might not represent current tobacco use prevalence, awareness, and knowledge. Finally, the indicator assessing knowledge of the risks for tobacco simultaneously inquired about both respondents’ knowledge and beliefs related to each outcome; thus, it was not possible to differentiate between these two constructs.

Although overall knowledge that smoking causes lung cancer, heart attack, and stroke is relatively high in most countries, opportunities exist to increase this knowledge across all countries and populations, including among current tobacco smokers. Implementation of the evidence-based measures outlined in the WHO FCTC and MPOWER, which include mass media campaigns and graphic health warning labels on tobacco products, can increase knowledge that smoking causes noncommunicable diseases such as chronic disease, including stroke, heart attack, and lung cancer. Increasing knowledge about the risks posed by tobacco smoking could help curb the estimated 1 billion tobacco-related deaths projected to occur in the 21st century (*3*–*5*,*7*,*9*).

**Summary**

**What is already known about this topic?**

Smoking is a leading preventable cause of disease globally, and increasing knowledge of the health effects of smoking can help to decrease smoking and increase successful cessation.

**What is added by this report?**

Across 28 countries, the median prevalences of tobacco smoking and smokers making a quit attempt were 22.5% and 42.5%, respectively. The median prevalences of knowing that tobacco smoking causes stroke, heart attack, and lung cancer were 73.6%, 83.6%, and 95.2%, respectively.

**What are the implications for public health practice?**

Implementation of proven tobacco control interventions, including strategies that increase knowledge about the health risks of tobacco use, could reduce tobacco use and tobacco-related diseases, including stroke, heart attack, and lung cancer.

## Footnotes

* The definition of smoking did not include electronic cigarettes, noncombustible tobacco products, and newer product such as heated tobacco products (referred to as “heat-not-burned” by the tobacco industry).

† Article 12 of the WHO FCTC states “Each Party shall promote and strengthen public awareness of tobacco control issues, using all available communication tools, as appropriate. Toward this end, each Party shall adopt and implement effective legislative, executive, administrative or other measures to promote: (b) Public awareness about the health risks for tobacco consumption and exposure to tobacco smoke, and about the benefits of the cessation of tobacco use and tobacco-free lifestyles as specified in Article 14.2.; (f) Public awareness of and access to information regarding the adverse health, economic, and environmental consequences of tobacco production and consumption.” http://apps.who.int/iris/bitstream/handle/10665/42811/9241591013.pdf;jsessionid=F1A7FFF03B5BF40033AEBE82E1590520?sequence=1.

§ The six components of MPOWER are “monitor” tobacco use and prevention policies; “protect” people from tobacco smoke; “offer” help to quit tobacco use; “warn about the dangers of tobacco; “enforce” bans on tobacco advertising, promotion, and sponsorship; and “raise” taxes on tobacco. http://www.who.int/tobacco/mpower/mpower_report_full_2008.pdf.

¶ Sustainable Development Goal 3 — Good Health and Well-Being states “Ensuring healthy lives and promoting the well-being for all at all ages is essential to sustainable development… However, many more efforts are needed to fully eradicate a wide range of diseases and address many different persistent and emerging health issues.” https://www.un.org/sustainabledevelopment/health/.
